# Exploring the Mechanisms of Testicular Aging: Advances in Biomarker Research

**DOI:** 10.14336/AD.2025.0070

**Published:** 2025-03-07

**Authors:** Wenkang Chen, Hede Zou, Haoran Xu, Rui Cao, Yapeng Zhang, Yongjie Ma, Wei Lin, Hekun Zhang, Jiayou Zhao

**Affiliations:** ^1^Graduate School of China Academy of Chinese Medical Sciences, Beijing, China.; ^2^Graduate School of Hebei University of Chinese Medicine, Shijiazhuang, China.; ^3^Institute of Basic Research in Clinical Medicine, China Academy of Chinese Medical Sciences, Beijing, China.

**Keywords:** testicular aging, biomarkers, mechanisms, male fertility, clinical translation

## Abstract

Aging biomarkers quantify aging progression and provide actionable targets for therapeutic interventions to mitigate age-related decline. This review synthesizes emerging evidence on testicular aging biomarkers, focusing on cellular senescence (Leydig, Sertoli, and endothelial cells), protein homeostasis disruption, mitochondrial dysfunction, germ stem cell depletion, sperm telomere length, epigenetic alterations, oxidative stress, inflammation, and gut microbiota dysbiosis. We propose that testicular aging serves as a critical nexus linking reproductive decline with systemic aging processes, with its pathological progression being quantifiable through specific biomarkers including the Leydig, Sertoli, and endothelial cells, *INSL3*, ribosomal protein *RPL39L*, sperm telomere length, relative telomere length mitochondrial translocator protein, and sialic acid. By bridging systemic aging paradigms with testis-specific mechanisms, we emphasize the urgency to identify organ-selective biomarkers for targeted interventions, advancing strategies to preserve male fertility and address population aging challenges.

## Introduction

Global population aging has escalated the burden of age-related chronic diseases, driven by progressive functional decline across cellular, molecular, and systemic levels [1]. Aging involves functional and structural degeneration across cellular, protein, and genetic levels. As conceptualized by López-Otín et al [2,3], aging biomarkers serve as dynamic indicators of physiological decline and potential levers for therapeutic modulation. These biomarkers not only serve as indicators of aging severity but also accelerate the aging process and can act as potential targets for interventions aimed at mitigating aging. Aging biomarkers reflect biological changes across multiple dimensions, encompassing the decline of cellular, tissue, and organ function.

Research demonstrates a continuous decline in male fertility [4], and the testes, as one of the key male reproductive organs, reproductive aging impairs fertility [5]. Research indicates that compared to younger men, elderly men show damage to Leydig cells (LCs) and Sertoli cells (SCs), with significant abnormalities in spermatogenesis and sperm quality [6]. While reproductive aging universally impairs fertility, testicular aging uniquely intersects with broader health crises. Beyond impairing fertility, testicular aging exacerbates systemic pathologies such as insulin resistance and cardiovascular dysfunction, positioning it as a nexus for holistic male health management [7,8]. However, current research predominantly focuses on systemic aging mechanisms and reproductive dysfunction, neglecting the organ-specific interplay and clinical translation between testicular decline and systemic deterioration.

**Figure 1.**
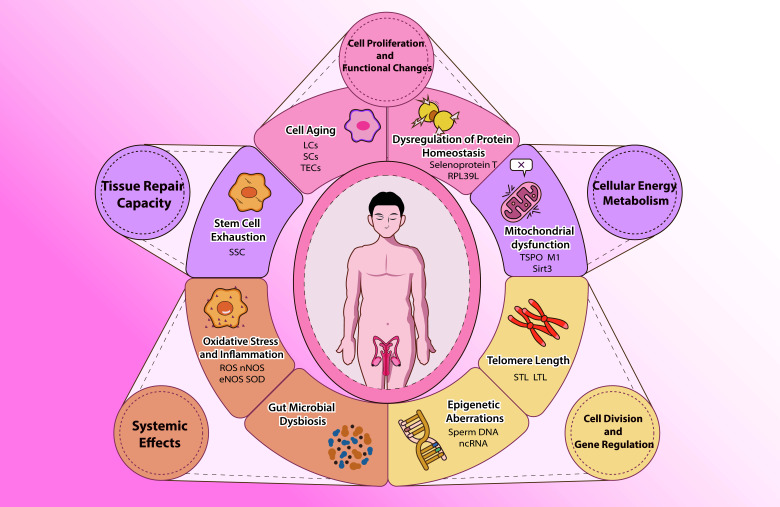
**Testicular Aging and Manifestations**. As depicted in the figure, testicular aging involves changes across five major areas: cellular proliferation and functional alterations, cellular energy metabolism, cell division and gene regulation, tissue repair capacity, and systemic effects. Specifically, testicular aging is characterized by the testicular cells aging, including Leydig, Sertoli, and endothelial cells. Other manifestations include dysregulation of protein homeostasis, mitochondrial dysfunction, spermatogonial stem cell exhaustion, sperm telomere Length, testicular epigenetic alterations (such as sperm DNA methylation and non-coding RNAs), oxidative stress, inflammation, and dysbiosis of the gut microbiota, among other systemic effects.

Drawing on the paradigms of aging biomarkers research, this review addresses critical gaps by integrating testis-specific biomarkers (e.g., *INSL3*, sperm telomere length, Testis-specific ribosomal protein *RPL39L*, Sialic acids) with systemic aging pathways. as shown in Figure 1, exploring potential testicular specific aging biomarkers and their underlying mechanisms, and providing research directions and references for early diagnosis and intervention strategies in testicular aging.

### Cell Aging and Testicular Aging

**Figure 2.**
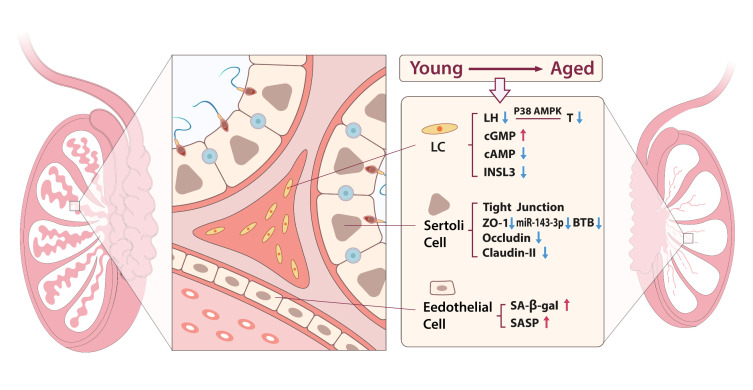
**Testicular cell aging**. During aging, testicular cell aging primarily involves testicular Leydig, Sertoli, and endothelial cells. Senescent testicular LCs exhibit a decrease in LH levels, which, through p38 MAPK signaling, leads to a reduction in testosterone levels. In these senescent LCs, cGMP levels increase while cAMP levels decrease, and *INSL3* expression is diminished. In senescent testicular SCs, a decline in tight junctions, ZO-1, occluding, and claudin-11 occurs, leading to damage to the BTB. Furthermore, senescent testicular endothelial cells show positive SA-β-gal staining, along with increased expression of genes associated with the SASP.

Cellular senescence is a fundamental mechanism of organismal aging, and arises from cumulative stressors including oxidative damage, autophagic dysfunction, and genomic instability, collectively disrupting cell cycle regulation [9]. Senescent cells drive tissue dysfunction via the senescence associated secretory phenotype (SASP), characterized by proinflammatory cytokine release, which perpetuates chronic inflammation and impairs regenerative potential [10]. In testes, senescence of LCs, SCs, and testicular endothelial cells (TECs) synergistically undermine reproductive and endocrine functions(Fig. 2).

LCs, the primary androgen-producing cells in testes, orchestrate spermatogenesis through testosterone synthesis and paracrine signaling to adjacent SCs [11,12]. Aging disrupts LH responsiveness via cyclic nucleotide imbalance (elevated cGMP, diminished cAMP), blunting testosterone output [13]. Concurrent p38 MAPK activation exacerbates LC degeneration, as evidenced by oxLDL-induced suppression of StAR and P450scc—key enzymes in steroidogenesis [14,15]. Notably, *INSL3*(insulin-like factor 3), a biomarker of LC differentiation, declines by ~15% per decade post-age 35, correlating with LC depletion and functional impairment [16,17]. In elderly men, *INSL3* levels negatively correlate with LC numbers, and *INSL3* mRNA expression correlates with both age and LC quantity changes. Therefore, *INSL3* can be considered a valuable biomarker for assessing the aging of testicular interstitial cells [18-20].

SCs play a crucial role in spermatogenic support, maintaining the blood-testis barrier (BTB), regulating the microenvironment of sperm development, and aiding spermatogenesis. Studies show that, with aging, tight junctions (TJs) formed by adjacent supporting cells gradually degrade, accompanied by ultrastructural abnormalities. The expression of tight junction proteins, such as zonula occludens-1 (ZO-1), occludin, and claudin-11, decreases, resulting in impaired tight junction barrier function and degeneration of seminiferous tubules. This may be driven by p38 MAPK/MMP9 pathway activation and autophagy dysfunction in SCs [21]. Moreover, studies suggest that *miR-143-3p* further accelerates SC senescence by targeting *UBE2E3* (ubiquitin-conjugating enzyme E2E3), compromising BTB integrity; its inhibition restores barrier function, and alleviates SC senescence, highlighting therapeutic potential [22].

TECs supply blood and nutrients to the testes and cooperate with other testicular cells to maintain the testicular microenvironment. And form a vascular niche critical for nutrient delivery and spermatogonial stem cell (SSC) maintenance. Aging TECs display SA-β-gal positivity and SASP upregulation, impairing SSC proliferation and exacerbating germline attrition. For example, TECs senescence may lead to reduced spermatogenesis in aging males [23]. Notably, TECs transplantation rescues spermatogenesis in SSC-depleted models, underscoring their regenerative role [24].

While Leydig and Sertoli cell senescence are well-documented, their synergistic dysfunction remains understudied. For instance, LC-derived testosterone decline may impair SC-mediated BTB integrity, while SC senescence further disrupts LC paracrine support, creating a vicious cycle of testicular functional decay. Moreover, the translational relevance of biomarkers like *INSL3* requires validation in longitudinal clinical cohorts to establish causality beyond correlation. Regarding testicular cell aging, future studies should prioritize multi-omics approaches to map cross-cellular communication networks in aging testes. Targeting shared pathways (e.g., p38 MAPK) or SASP components may offer dual therapeutic benefits for Leydig, Sertoli, and endothelial cell senescence.

### Protein Homeostasis Dysregulation and Testicular Aging

Protein homeostasis refers to the dynamic balance of the cellular proteome, which is maintained through processes such as synthesis, folding, modification, and degradation [25,26]. During aging, key drivers include impaired autophagy, ribosomal dysfunction, and endoplasmic reticulum (ER) stress, culminating in toxic protein aggregation and functional decline [27]. As aging progresses, the capacity of cells to preserve protein homeostasis declines, leading to an imbalance characterized by suppressed transcription of heat shock and unfolded protein responses. This imbalance promotes protein aggregation and abnormal protein folding and degradation [28].

Ubiquitination, a post-translational modification, is essential for regulating key physiological functions, including protein degradation. The ER quality control system utilizes the ubiquitin-proteasome pathway to recognize and degrade unnecessary proteins within the ER [29]. Selenoprotein T, an ER-resident redox regulator, safeguards Leydig cell steroidogenesis by maintaining ER proteostasis. Its age-related decline correlates with oxidative stress and mitochondrial dysfunction [30-32], though its testis-specific regulatory networks remain undefined. Furthermore, the testis-enriched ribosomal protein *RPL39L* is critical for murine spermatogenesis and protein homeostasis. Its deletion impairs spermatogonial stem cell proliferation, and induces mitochondrial and flagellar abnormalities in sperm, while lacking human homolog validation, highlighting a key translational gap [33,34].

### Mitochondrial Dysfunction and Testicular Aging

Mitochondria regulate energy metabolism through tightly coupled ATP synthesis and reactive oxygen species (ROS) generation. Aging disrupts this balance, with declining respiratory capacity and ROS overproduction driving cellular senescence [35]. Mitochondria are closely linked to the aging process, regulated by nutrient sensors such as mTOR, AMPK, and sirtuins. Through autophagy, they modulate mitochondrial fusion, fission, and turnover, thereby controlling mitochondrial biogenesis and dynamics [36]. Mitochondrial dysfunction, characterized by reduced respiratory capacity and membrane potential, is a key cause and marker of cellular aging, playing a crucial role in inducing and maintaining aging phenotypes [37].

In LCs, mitochondrial dysfunction impairs cholesterol transport (via translocator protein [TSPO] downregulation) and steroidogenic enzyme activity (e.g., steroidogenic acute regulatory protein [StAR]), reducing testosterone synthesis. Pharmacological modulation of cGMP signaling (e.g., PDE5 inhibitors) rescues mitochondrial dynamics and testosterone synthesis in aged LCs. Studies suggest that chronic use of PDE5 inhibitors can restore mitochondrial function, dynamics, and testosterone synthesis by reducing NO and cGMP levels [38,39]. Moreover, activation of the mitochondrial fusion promoter M1 has been shown to enhance mitochondrial function in aged LCs, improving their activity and promoting testosterone synthesis [40]. In addition, a study by Xi H et al [41]. found that Trehalose, an autophagy inducer, activates testicular autophagy, upregulates tight junction protein ZO-1 expression, enhances mitochondrial clearance in SCs, highlighting the therapeutic potential of organelle-targeted interventions.

**Figure 3.**
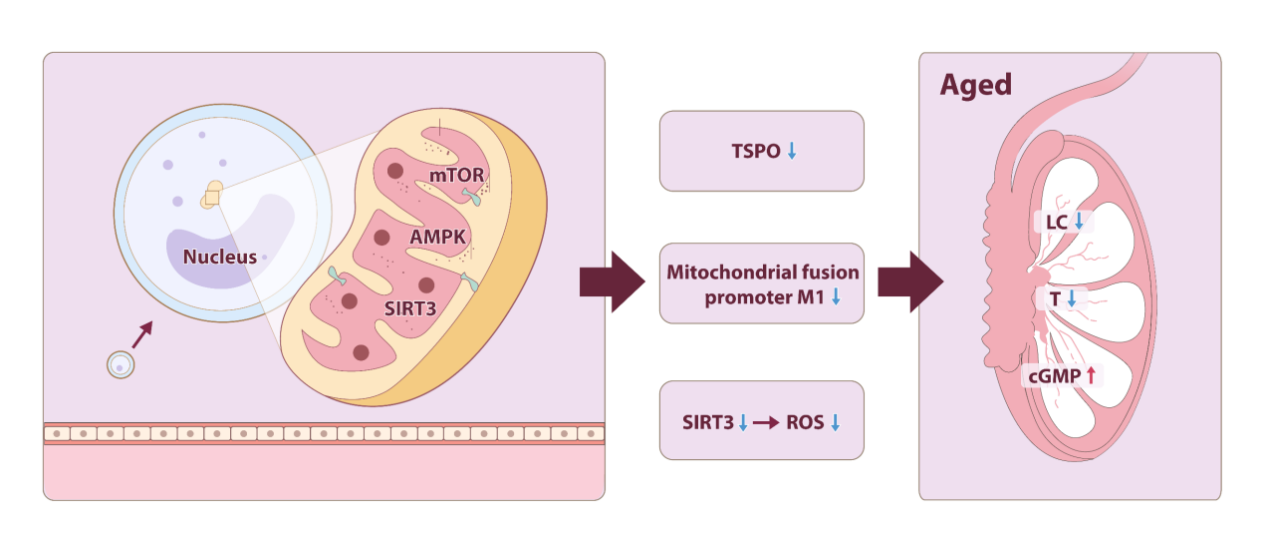
**Testicular Aging and Mitochondrial Dysfunction**. Mitochondrial dysfunction contributes to testicular aging through three key pathways: mTOR, AMPK, and sirtuins. During this process, the levels of TSPO, mitochondrial fusion protein M1, and *SIRT3* decreased, leading to a reduction in the number of LCs, lower T levels, and an increase in cGMP levels within the testes.

*SIRT3*, an NAD+-dependent mitochondrial deacetylase, exhibits conflicting roles in testicular aging: while ovarian *SIRT3* declines with age, murine testicular *SIRT3* remains stable but susceptible to environmental toxins (e.g., 1-nitropyrene). This discrepancy underscores the need for species- and context-specific analyses [42,43]. *SIRT3* knockdown in LCs elevates ROS and disrupts progesterone synthesis, whereas its activation via MitoQ mitigates toxin-induced senescence, suggesting context-dependent therapeutic utility (Fig. 3). The role of *SIRT3* in mitochondrial function and testicular aging requires further investigation [44].

### Stem Cell Exhaustion and Testicular Aging

Spermatogonial stem cells (SSCs) serve as the cornerstone of tissue homeostasis and regenerative capacity across multiple organ systems, including the testis. The exhaustion of SSCs is a hallmark of aging, characterized by a decline in regenerative capacity [45]. Phenotypic hallmarks include cell cycle arrest, β-galactosidase activation, and mitochondrial dysfunction [46]. These features are shared across somatic stem cell types but uniquely exacerbated in testes by gonad-specific stressors (e.g., BTB breakdown). SSCs, essential for sustained spermatogenesis, progressively decline with age due to oxidative damage, epigenetic dysregulation, and pro-inflammatory microenvironmental shifts [47,48]. Single-cell transcriptomics reveals age-related skewing of SSCs toward differentiation-committed states in rodents, accompanied by DNA hypomethylation and 5-hydroxymethylcytosine (5hmC) accumulation [49,50]—a pattern tentatively linked to human oligozoospermia but requiring clinical validation.

Research by Zhang et al [51]. demonstrated that in aged mouse testes, the balance between undifferentiated and differentiated spermatogonial stem cells is disrupted, alongside an increase in age-specific macrophage subpopulations, which may exacerbate the pro-inflammatory microenvironment during testicular aging. Stem cell transplantation, such as bone marrow mesenchymal stem cells (BMSC), testicular stromal stem cells, and umbilical cord mesenchymal stem cells, has emerged as a potential new strategy for treating testicular aging [52]. However, there is also a potential risk of immune rejection and poor engraftment in clinical translation. Small-molecule senolytics (e.g., navitoclax) targeting β-galactosidase-positive cells may offer safer alternatives [46]. Glutathione supplementation demonstrates preclinical efficacy in reversing SSC oxidative damage [53], yet its efficacy in human trials remains to be confirmed.

### Telomere Length and Testicular Aging

Telomeres are composed of repetitive TTAGGG sequences and telomere-binding proteins, located at the ends of chromosomes. They maintain chromosomal integrity and stability by preventing degradation and fusion of chromosomal ends [54]. As individuals age, telomeres progressively shorten, and their length is influenced by genetic, disease, and environmental factors. Telomere shortening accompanies cell division, and critically short or dysfunctional telomeres may trigger a DNA damage response, leading to cellular replicative senescence [55]. Telomeres are rich in guanine, making them susceptible to oxidative modifications that lead to replication arrest and shortening. Lifestyle factors (e.g., smoking, obesity) exacerbate telomere shortening via ROS-mediated guanine oxidation, while exercise and antioxidants (e.g., vitamin E) exert protective effects [56-58].

Sperm telomere length (STL) is an important biomarker of spermatogenesis and sperm quality [59]. Studies have shown that STL is positively correlated with sperm motility and viability, negatively correlated with DNA fragmentation, and closely associated with mitochondrial DNA abnormalities [60,61]. Sperm telomeres paradoxically elongate with advancing age, contrasting leukocyte telomere attrition, this divergence may reflect germline-specific telomerase activity [62,63]. Furthermore, studies have revealed a positive correlation between sperm and leukocyte telomere length, sperm concentration, and total count [64]. Although STL increases chronologically, its functional significance remains debated and requires further research. In patients with oligoasthenoteratozoospermia, both sperm and leukocyte telomere lengths are significantly shortened, and shortened sperm and leukocyte telomeres correlate with seminal *SIRT1/3* deficiency, suggesting systemic redox dysregulation as a potential mechanism [65]. Study suggests that STL holds diagnostic and prognostic value for male fertility and clinical pregnancy outcomes, and may also serve as a biomarker for male infertility and embryo development prediction [66].

Moreover, male germ cells uniquely retain telomerase activity to offset replicative telomere shortening, yet ROS and inflammation during epididymal maturation induce age-dependent relative telomere length (rTL) attrition, positioning rTL as a dynamic biomarker of testicular aging [67]. Standardized assays (e.g., single-telomere length analysis) are urgently needed to resolve conflicting clinical associations and establish STL/rTL thresholds for infertility diagnosis.

### Epigenetic aberrations and Testicular Aging

Epigenetic dysregulation, a hallmark of aging, encompasses DNA methylation shifts, histone modifications, and non-coding RNA (ncRNA) alterations [68]. These changes drive testicular aging phenotypes, including stem cell exhaustion and mitochondrial dysfunction, yet their testis-specific regulatory networks remain poorly characterized.

DNA methylation at specific loci can serve as an epigenetic clock, providing an accurate prediction of an individual's biological age [69]. Sperm DNA methylation landscapes function as epigenetic clocks, with hypermethylation at developmental loci-correlating with reduced fertility in aging males [68]. The aging process is accompanied by epigenetic modifications and mutations in sperm DNA, and these epigenomic alterations are profound, dynamic, and potentially irreversible. Such modifications may affect paternal reproductive health and be passed on to offspring through epigenetic information [70]. Studies have demonstrated significant age-dependent changes in the sperm DNA methylome, with environmental exogenous factors potentially disrupting the sperm epigenetic aging process [71]. For example, abnormal DNA methylation in the testes during aging can be restored by young plasma transplantation, which rejuvenates the testicular epigenome and increases spermatogonia and sperm counts [72]. While young plasma transplantation reverses murine testicular methylation aging, its clinical translatability and ethical implications require rigorous evaluation.

The mTOR pathway, a central target in both aging and anti-aging research, is highly expressed in the testes and regulates the integrity and permeability of the BTB. The mTOR pathway exhibits dual roles in testicular epigenetics: mTORC2 promotes sperm DNA hypermethylation, whereas mTORC1 enhances TET-mediated demethylation. This dichotomy suggests that pathway-selective therapeutics (e.g., rapamycin analogs) could rejuvenate the aging male germline by fine-tuning methylation dynamics [73,74].

**Figure 4.**
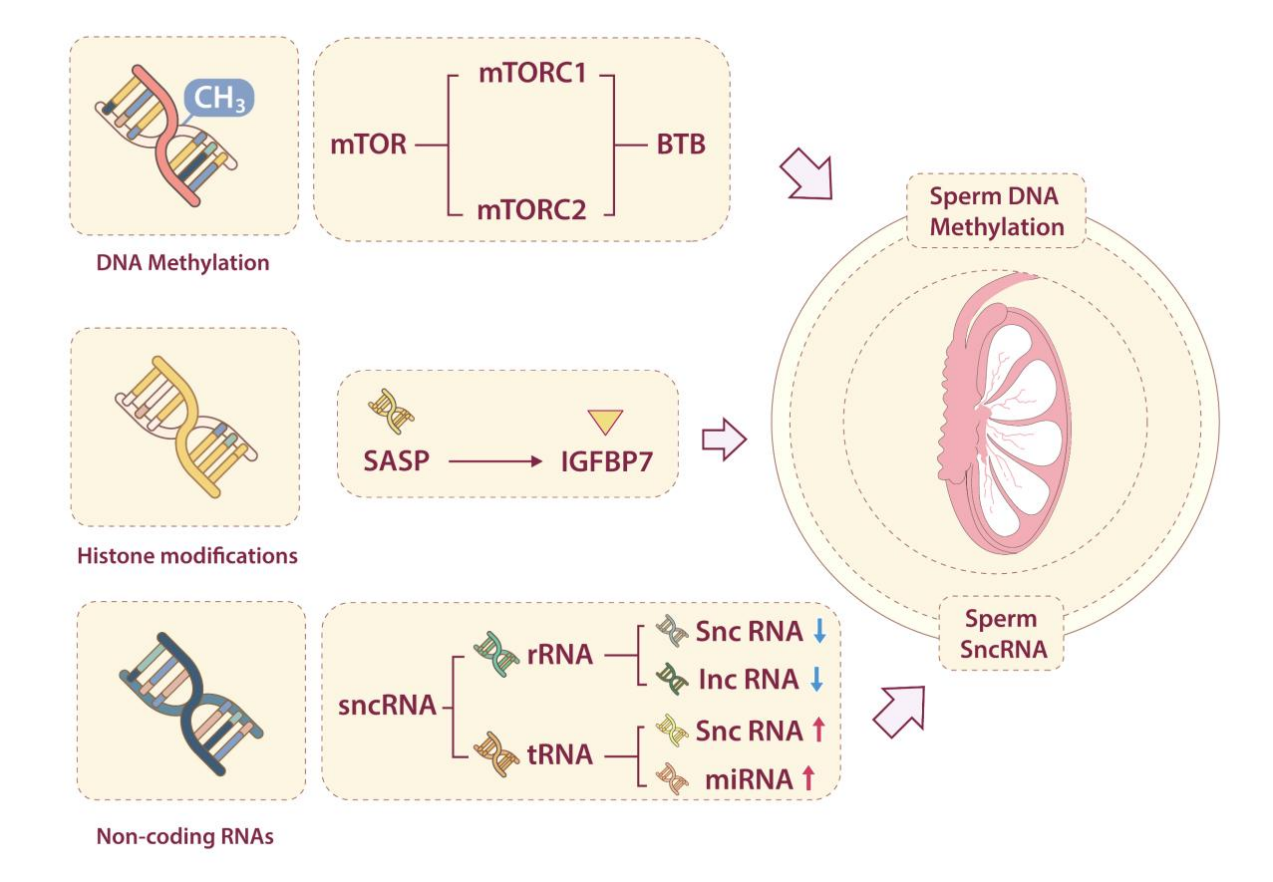
**Epigenetic Aberrations and Testicular Aging**. Epigenetic aberrations, including DNA methylation, histone modifications, and ncRNAs, contribute to testicular aging. These alterations can influence the integrity of the BTB via the mTOR signaling pathway, specifically through mTORC1 and mTORC2, sperm DNA methylation in aging testes is affected. Additionally, the levels of sncRNAs change with testicular aging. Specifically, levels of rRNA-derived sncRNAs and lncRNAs decrease, while tRNA-derived sncRNAs and miRNAs levels increase as the testis aging.

Aging also profoundly affects the small non-coding RNA (sncRNA) profile of sperm. Studies have demonstrated age-dependent changes in the composition of sperm sncRNAs in rats. Specifically, the proportions of rRNA-derived sncRNAs and lncRNAs decrease with age, whereas tRNA-derived sncRNAs and miRNAs increase. Notably, piRNAs remain stable with age, suggesting their role in maintaining genomic stability may buffer against epigenetic drift [75]. Sperm sncRNAs can mediate the intergenerational transmission of acquired paternal phenotypes. For instance, alterations in sperm sncRNA content induced by a high-fat diet are associated with changes in offspring phenotypes, such as insulin resistance, altered body weight, and impaired glucose tolerance [76,77].

Additionally, research has identified that *IGFBP7*, a SASP factor elevated in aged testes, disrupts Sertoli-germ cell communication via integrin signaling (Fig. 4).

Despite its biomarker potential for testicular aging, *IGFBP7*’s causal role in human infertility and its interplay with epigenetic mechanisms remains to be confirmed [78].

### Oxidative Stress, Inflammatory Response, and Testicular Aging

The accumulation of ROS can damage nucleic acids and proteins, alter cellular structures and functions, impair antioxidant defense mechanisms, and increase cytotoxicity, thereby accelerating aging [79]. During the aging process, inflammatory responses typically center around the vascular system, with an increase in the expression of pro-inflammatory cytokines, activation of inflammatory signaling, and accumulation of senescent cells, which exacerbate the levels of inflammation [80]. Oxidative stress arises from an imbalance between ROS production and antioxidant mechanisms, leading to chronic inflammation. ROS can activate various transcription factors, triggering changes in the expression of inflammation-related genes [81]. The interplay between oxidative stress and inflammation collectively contributes to aging and the development of age-associated diseases.

ROS overproduction in aging testes disrupts redox homeostasis, inducing nucleic acid damage, protein misfolding, and lipid peroxidation. Concurrently, senescent cell accumulation fuels a pro-inflammatory milieu via NF-κB and NLRP3 inflammasome activation, creating a vicious cycle that exacerbates testicular dysfunction. Aging SCs exhibit impaired antioxidant defenses (e.g., reduced *SOD1* and catalase), leading to ROS-driven BTB disintegration via the downregulation of tight junction proteins (ZO-1, claudin-11). This breach permits immune cell infiltration, amplifying local inflammation and germ cell apoptosis [82,83]. Notably, macrophage-derived IL-1β further suppresses Leydig cell steroidogenesis, linking inflammation to hormonal decline. Melatonin, which has anti-inflammatory and antioxidant properties, directly and indirectly regulates testicular aging through the hypothalamic-pituitary axis. It reduces oxidative stress and inflammation, thereby improving testicular function, including steroidogenesis and spermatogenesis [84-86]. Natural antioxidants (e.g., wild berry polyphenols) show preclinical promise to mitigate inflammation and oxidative stress in aging testes but lack standardized formulations for clinical translation [87].

Furthermore, Age-related nicotinamide adenine dinucleotide (NAD+) depletion impairs sirtuin-mediated deacetylation (e.g., *SIRT1*), disrupting spermatogonial differentiation and steroidogenesis [88]. Studies have shown that during the aging process, the levels of NAD in both blood and testicular tissues gradually decline. Male mice with NAD deficiency exhibit testicular atrophy, impaired spermatogonia proliferation and differentiation, and reduced sperm count. After NAD supplementation, testicular weight, and sperm quality are restored, suggesting that NAD may serve as a potential biomarker for testicular aging [89]. Although NAD+ precursors restore murine spermatogenesis, their long-term safety and efficacy in humans require validation. In addition, it is necessary to elucidate how NAD+ depletion intersects with epigenetic dysregulation in driving testicular senescence.

### Gut Microbial Dysbiosis and Testicular Aging

Gut microbes can induce senescence in ileal B cells and reduce the diversity of immunoglobulin A (IgA) antibodies, leading to microbial imbalance. Through the host immune system, the gut microbiota and cellular senescence form a vicious cycle that further disrupts the age-related balance of the gut microbiome [90,91]. Emerging evidence highlights the gut-testis axis as a bidirectional regulator of male reproductive aging. The gut microbiota interacts bidirectionally with the testes through metabolic products (such as short-chain fatty acids, SCFAs, and bile acids), immune regulation, and hormone signaling pathways [92,93]. Age-related gut dysbiosis disrupts microbial metabolite production (e.g., SCFAs), impairing systemic immune homeostasis and exacerbating testicular senescence. Specific taxa depletion (e.g., *Lactobacillus*) correlates with reduced serum testosterone and sperm quality [94].

Fecal microbiota transplantation (FMT) from young donors restores spermatogenesis in aged mice via microbial metabolites (e.g., 3-hydroxyphenylacetic acid), potentially inhibiting germ cell ferroptosis through GPX4 upregulation [95]. In aging boars, alginate oligosaccharides (AOS) increase *Enterobacteriaceae* abundance and serum testosterone [96], yet human trials must validate these effects and address safety concerns (e.g., pathogen overgrowth).

Targeted modulation of gut microbiota represents a promising therapeutic strategy for delaying testicular senescence. Future research should prioritize multi-omics integration (e.g., single-cell transcriptomics and metabolomics) to delineate the spatiotemporal regulatory dynamics of the gut-testis axis during aging, particularly focusing on how specific microbial metabolites (e.g., 3-hydroxypropionic acid) traverse the BTB to directly interact with testicular cell populations.

### Other Biomarkers: Glycosylation and Peptide Signaling

Beyond classical biomarkers, testicular hyposialylation—marked by declining sialic acid (Sia) levels—disrupts glycoprotein-mediated cell adhesion, impairing sperm maturation. Sialylation status, regulated by sialyltransferases (e.g., *ST6GAL1*), serves as a dynamic biomarker of testicular aging. Murine models show *ST6GAL1* downregulation with age [97,98], but human correlative studies are lacking, necessitating longitudinal proteomic analyses.

Concurrently, the relationship between peptide substances and testicular aging has been increasingly recognized. Age-dependent adiponectin deficiency in LCs exacerbates insulin resistance and steroidogenic decline. Adiponectin receptor agonists rescue spermatogenesis in mice, yet their efficacy in humans and interplay with epigenetic regulators (e.g., *SIRT1*) remain unexplored [99,100].

**Table 1:** Hallmarks of Testicular Aging.

		Hallmarks of Testicular Aging	Changing Trends	Reference
Cell Aging	Leydig cells	testosterone (T)	decrease	[11,12]
		**Luteinizing hormone (LH)**	decrease	[14,15]
		**cGMP levels in LC**	increase	[38,39]
		**cAMP levels in LC**	decrease	[13]
		**p38 MAPK in LC**	activated	[21]
		**Insulin-like factor 3(*INSL3*)**	decrease	[16,17,18,19,20]
	Sertoli cells	Tight Junction(TJ)	destroyed	[21]
		**zonula occludens-1 (ZO-1)**	decrease	[21,41]
		**occludin**	decrease	[21]
		**claudin-11**	decrease	[21]
	Testicular endothelial cells	Testicular endothelial cells (TECs)	decrease	[23,24]
		**SA-β-gal**	positive	[23]
**Dysregulation of Protein Homeostasis**		Selenoprotein T	decrease	[30,31,32]
		**Testis-specific ribosomal protein *RPL39L***	decrease	[33,34]
**Stem Cell Exhaustion**		Somatic stem cells	gradual exhaustion	[45,46,47,48,51]
**Telomere Length**		Sperm telomere length (STL)	shorten	[60,61,62,63,64,65]
		**Leukocyte Telomere Length (LTL)**	shorten	[64]
		**Sperm relative telomere length (rTL)**	shorten	[67]
**Epigenetic Aberrations**		Methylation level of sperm DNA	decrease	[68,71,72]
		**mTORC1**	negative correlation	[73,74]
		**mTORC2**	positive correlation	[73,74]
		**sncRNA**	decrease	[75,76,77]
		**lncRNA**	decrease	[75]
		**miRNA**	increase	[75]
		***IGFBP7***	increase	[78]
**Mitochondrial dysfunction**		TSPO	decrease	[38,39]
		**Mitochondrial fusion promoter M1**	decrease	[40]
		**Sirtuin-3 (*SIRT3*)**	decrease	[42,43,44]
**Oxidative Stress and Inflammation**		ROS	increase	[79,81]
		**Superoxide dismutase (SOD) activity**	decrease	[82,83]
		**Tight junction protein and adhesion junction protein of BTB**	decrease	[83]
		**Melatonin levels**	decrease	[84,85,86]
		**nicotinamide adenine dinucleotide (NAD)**	decrease	[88,89]
**Gut Microbial Dysbiosis**		Needs further study	
Other Biomarkers		Sialic acids(Sia)	decrease	[97,98]
		**Adiponectin**	decrease	[99,100]

### Potential Intervention Strategies

Emerging interventions targeting testicular aging demonstrate therapeutic potential for decelerating reproductive system degeneration. In pharmacological approaches, Preclinical studies demonstrate that senolytic agents selectively eliminating senescent cells extend murine lifespan while reducing senescence-associated β-galactosidase activity [101,102]. The combination of dasatinib and quercetin enhances serum testosterone levels, improves sperm concentration, and ameliorates sperm morphological abnormalities in aging models [103]. NAD+ precursor supplementation (e.g., NMN, NR) restores *SIRT1/3*-dependent mitochondrial function in aged LCs, enhancing testosterone biosynthesis while attenuating oxidative stress [44,104,105]. Epigenetic modulators including DNA methyltransferase (DNMT) inhibitors (e.g., 5-azacytidine) [106] and TET enzyme activators (e.g., vitamin C) effectively rejuvenate sperm DNA methylation patterns in senescent rodents [107,108], demonstrating the potential for targeted epigenetic regulation.

Breakthroughs in nanomedicine enable precision delivery: Research has shown that nanoparticle-mediated testicular delivery systems can penetrate the BTB and enhance testicular targeting [109-112]. Perhaps nanoparticle encapsulated senolytics, NMN, DNMT, and other drugs can be used as testicular targeted drug delivery systems to accurately target and eliminate aging testicular cells, restore self-renewal of testicular cells, and maintain the integrity of BTB. Intriguingly, gut-testis axis modulation emerges as a novel paradigm [92,113]—study indicate gut microbiota modulation through FMT from young donors or *Lactobacillus*-dominant probiotics reverses age-related dysbiosis, elevates serum testosterone [114], and reduces testicular inflammation via microbial metabolites like 3-hydroxyphenylacetic acid [95], yet mechanistic details remain to be studied and clarified.

In addition to classic strategies targeting aging mechanisms, traditional medicine exhibits unique anti-aging properties through multi-target mechanisms [115,116], warranting further investigation. Moreover, despite promising animal data, it warrants rigorous safety evaluation before clinical translation due to species-specific differences.

### Conclusions and Future Perspectives

Testicular aging represents a pivotal interface between reproductive decline and systemic aging, driven by organ-specific biomarkers including LCs, SCs, TECs, *INSL3*, protein *RPL39L*, STL, rTL, TPSO, Sia, and sperm telomere dynamics. These biomarkers intersect with critical signaling pathways governing cellular proliferation, functional adaptation, energy metabolism, mitotic regulation, genomic stability, and tissue regenerative capacity. While these biomarkers share mechanistic links with generalized aging processes (e.g., Cellular senescence, Protein Homeostasis Dysregulation, mitochondrial dysfunction, epigenetic drift), their organ-enriched expression and functional specificity in testes offer unique diagnostic and therapeutic opportunities. Strategic targeting of these biomarkers holds dual potential for preserving fertility and mitigating age-related comorbidities, positioning testicular health as a cornerstone of male healthy aging strategies. However, there are still some critical gaps in current research including: The predominant reliance on rodent models for studying biomarkers like *RPL39L* and TSPO raise concerns about clinical translatability; The testis-specific aging markers including *IGFBP7*, *INSL3*, Sia, etc, still need further research and validation.

To bridge these gaps, future clinical translation and research should prioritize: (1) Diagnostic innovation: Develop non-invasive tools for monitoring testicular aging, such as semen sncRNA panels and STL/rTL ratio assays; (2) Therapeutic advancement: Engineering testis-targeted drug delivery systems (e.g., nanoparticle-encapsulated senolytics) to enhance the targeting of interventions; (3) Mechanistic integration: Establishing testis-specific aging networks through multi-omics integration (proteome-metabolome-epigenome) in longitudinal human cohorts to identify nodal biomarkers. And elucidating multi-organ crosstalk mechanisms (testis-gut axis, testis-brain axis, testis-hepatic axis, etc.); (4) Interdisciplinary convergence: Fostering collaboration between geroscience, biomaterials engineering, nanotechnology, and andrology to accelerate the mechanism of testicular aging research and clinical translation.
